# School-level associations between health-related fitness, school characteristics, and academic performance among U.S. youth: an observational study

**DOI:** 10.1016/j.pmedr.2026.103595

**Published:** 2026-08-04

**Authors:** Timothy J. Walker, Andjelka Pavlovic, Christopher D. Pfledderer, Derek W. Craig, Kevin Lanza, Kempson Onadeko, Matthew Lee, Laura F. DeFina

**Affiliations:** a Center for Health Promotion and Prevention Research at the University of Texas Health Science Center at Houston School of Public Health, Houston, TX, United States; b Institute for Implementation Science at the University of Texas Health Science Center at Houston School of Public Health, Houston, TX, United States; c Kenneth H. Cooper Institute at the Texas Tech University Health Sciences Center, Dallas, TX, United States; d Department of Health Promotion & Behavioral Sciences at the University of Texas Health Science Center at Houston School of Public Health Austin Campus, Austin, TX, United States; e Department of Environmental and Occupational Health Sciences at the University of Texas Health Science Center at Houston School of Public Health Austin Campus, Austin, TX, United States

**Keywords:** Physical activity, Cardiorespiratory fitness, Body mass index, Student achievement, Educational outcome

## Abstract

**Objective:**

Examine school-level associations between school characteristics, health-related fitness, and academics.

**Methods:**

This observational study included schools in the NFL PLAY60 FitnessGram® project. We used 2022–2023 school year school-level data from three sources: 1) school characteristics data from the National Center for Education Statistics website; 2) cardiorespiratory fitness (CRF) and body mass index (BMI) data; and 3) academic data (students at or above grade-level for math and English Language Arts (ELA)) from state department of education websites. We used mixed effects linear regression models to examine aims.

**Results:**

One-hundred-sixty-six schools had CRF data and 67 had BMI data. Economic disadvantage was inversely associated with students meeting Healthy Fitness Zone (HFZ) BMI standards (β = −0.10, *p* < 0.001, R^2^ = 0.60), and academic performance (math, β = −0.38, p < 0.001, R^2^ = 0.52; ELA, β = −0.42, p < 0.001, R^2^ = 0.59). There were differences in the percentage of students meeting HFZ standards and academic performance across schools with different racial/ethnic compositions. School-level CRF was directly associated with academic performance (math, β = 0.10, *p* = 0.04, R^2^ = 0.52; ELA, β = 0.13, *p* = 0.006, R^2^ = 0.59), and there were no significant relations between BMI and academic performance (math, β = 0.26, *p* = 0.25, R^2^ = 0.54; ELA, β = 0.18, *p* = 0.47, R^2^ = 0.71).

**Conclusions:**

Findings highlight fitness and academic disparities related to economic disadvantage and race/ethnicity, and relations between fitness and academics.

## Highlights

• Economic disadvantage linked to lower fitness and academic performance.
• Cardiorespiratory fitness positively associated with reading and math achievement.
• Fitness and academic disparities observed across racial/ethnic school compositions.
• Findings support policies prioritizing student fitness for academics.

## 1 Introduction

Children's fitness is an important predictor of health outcomes later in life (Ruiz et al., 2009). Two critical components include cardiorespiratory fitness (CRF) and body composition. Low CRF and unfavorable body composition in childhood are risk factors for poor cardiometabolic health, cardiovascular disease, and poor mental health (Raghuveer et al., 2020; Marcus et al., 2022). Despite the documented benefits of maintaining healthy fitness levels, evidence indicates children's CRF has declined while body mass index (BMI) has increased in recent years, particularly during the COVID-19 pandemic (Pavlovic et al., 2025; Lange et al., 2021).

There is also evidence that children's CRF and BMI are associated with academic performance (Santana et al., 2017; Álvarez-Bueno et al., 2020; He et al., 2019). These relations have critical implications for schools given they serve as primary settings for physical activity and fitness promotion. Despite the importance of physical activity, fitness and academics, key disparities exist among schools. Schools serving under-resourced or racially/ethnically diverse communities often provide fewer physical activity opportunities (Thompson and London, 2023; Craig et al., 2024; Turner and Chaloupka, 2017), have lower fitness levels, and poorer academic performance compared to schools serving higher-income communities (Walker et al., 2020; White et al., 2016). While research examining links between fitness, demographics, and academics has contributed valuable insights, many of the existing studies are limited by small sample sizes focused on specific geographic regions (Santana et al., 2017; Álvarez-Bueno et al., 2020). Although school-level studies exist, research has mostly focused on individual-level links between demographics, fitness, and/or academics (Zhu et al., 2022; Bai et al., 2016).

CRF and BMI are commonly collected indicators of health-related fitness in schools. Although their assessments have limitations, they provide valuable information pertaining to the health risk of school communities. School-level analyses offer an important perspective given policies, practices, and interventions to improve fitness and learning outcomes are usually implemented at the school level (Walker et al., 2023). Further, school accountability ratings are often based on their school-level academic performance (Figlio and Loeb, 2011). Therefore, understanding school-level associations between fitness and academics is important. School-level analyses also account for community context, which is associated with both outcomes (Walker et al., 2020; White et al., 2016), and can inform school based interventions.

To address these gaps, this study examined school-level relations among health-related fitness, school characteristics, and academic performance in a sample of elementary, middle, and high schools across the U.S. The study objectives were to examine: 1) associations between school characteristics and health-related fitness; and 2) relations among health-related fitness, school characteristics, and academic performance.

## 2 Methods

### 2.1 Study design and population

This observational study used secondary data from the NFL PLAY 60 FitnessGram® Project (referred hereafter as NFL PLAY60FG) during the 2022–2023 academic year. NFL PLAY60FG is a nationwide initiative promoting physical activity and fitness in U.S. schools (Craig et al., 2024). This study used FitnessGram data and publicly available school characteristics and academic data, which we downloaded for the 2022–2023 school year from the National Center for Education Statistics (NCES) (National Center for Education Statistics (NCES) | IES, 2025) website and each state's department of education website, respectively, during the winter of 2024. We merged data using each school's unique NCES number. The Committee for the Protection of Human Subjects at UTHealth Houston School of Public Health approved the study (HSC-SPH-23-0098).

NFL PLAY60FG is available to elementary, middle, and high schools in public and charter systems. Schools are recruited into cohorts on an annual basis. The schools in this study began the program in the 2019–2023 school years (Cohorts 1–4). Inclusion criteria were schools needed: 1) CRF and/or BMI assessments completed in winter/spring (Jan-May) of 2023, and 2) accessible school characteristics and academic data. We excluded schools if <10% of their population had been tested for either CRF and/or BMI.

### 2.2 Measures

#### 2.2.1 FitnessGram data

We used the CRF and BMI variables from the criterion-referenced FitnessGram assessment (Morrow Jr et al., 2011). Physical education teachers at each school assessed student's CRF using the PACER test or a one mile run/walk test (Plowman and Meredith, 2013). Students' results were entered into a cloud-based data tracking platform (GreenLight Fitness, LLC), which calculates estimates of maximal oxygen consumption (milliliters per kilogram per minute) (Cureton et al., 1995; Morrow et al., 2010). Student results were classified into age- and sex-specific standards: 1) Healthy Fitness Zone™ (HFZ), 2) Needs Improvement, or 3) Needs Improvement-Health risk.

Schools assessed students' BMI by measuring and entering their height and weight into the platform, which uses standard calculations for BMI values (Morrow Jr et al., 2011; Plowman and Meredith, 2013). Students' BMI were classified into age- and sex-specific values associated with health benefits, and Centers for Disease Control standards and growth charts: 1) HFZ, 2) Needs Improvement, or 3) Needs Improvement-Health risk. We used school-level CRF and BMI values based on the percentage of students meeting HFZ standards out of the total number of students tested.

#### 2.2.2 Academic and school characteristics data

We downloaded 2022–2023 school-level academic data (the percentage of students performing at or above grade-level for English Language Arts (ELA) and math) along with their NCES numbers, school names, and addresses. If NCES numbers were not provided, we manually added the NCES numbers by matching school names and addresses.

We downloaded school characteristics from the NCES website: economic disadvantage (percentage of students eligible for free or reduced lunch), school enrollment (total students in the school), school level (i.e., elementary, middle, high), locale (city, suburban, town, rural), and school-level race/ethnicity (percentage of students who were White, Black, Hispanic, and another race). We created a school-level race/ethnicity variable by categorizing schools based on most students served: 1) majority White (≥50%); 2) majority Black (≥50%); 3) majority Hispanic (≥50%); majority another race (≥50%); and diverse (no single race/ethnic majority). Because most schools were elementary, we collapsed the school-level groupings to include elementary and non-elementary. Similarly, most schools were in cities or suburban areas, thus we collapsed the locale variable to city and non-city schools.

### 2.3 Statistical analysis

Because more schools collected CRF than BMI data, we created separate CRF and BMI samples. For each sample, we examined descriptive statistics, including means and standard deviations for continuous variables and frequencies with percentages for categorical variables. Next, we examined associations between school characteristics, and health-related fitness variables (CRF and BMI). To account for clustering by state, we examined the proportion of variance explained by state for CRF and BMI using intraclass correlation coefficients (ICCs). For CRF, the state-level variance was 0.10, indicating the need for multilevel models. We therefore used mixed-effects linear regression models, treating state as a clustering variable. We examined bivariate models first, then multivariable models that included school characteristic variables simultaneously, also controlling for cohort. For BMI, the variance explained by state was low <0.001. Thus, we used linear regression models to examine associations between school characteristics and BMI using the cluster-robust variance estimator. We examined both bivariate and multivariable models.

We followed similar procedures for testing associations between school characteristics, health-related fitness, and academic variables. We first examined variance explained by state for ELA and math variables using ICCs. The variance was 0.38 for ELA and 0.43 for math, thus we use mixed-effects linear regression models with CRF and school characteristics as independent variables, and ELA and math as dependent variables. We first examined bivariate models, then multivariable models that also controlled for cohort. We repeated this procedure with the BMI sample. For all models, we examined the proportion of variance explained (R^2^); for multilevel models, we calculated marginal and conditional values (Nakagawa and Schielzeth, 2013). We also examined potential interactions between economic disadvantage (using Title 1 eligibility defined as ≥40% of students from economically disadvantaged backgrounds) and each health-related fitness variable (CRF and BMI). We conducted all data preparation and modeling using StataSE 15.

## 3 Results

Of the participating schools, 195 conducted CRF assessments and 74 conducted BMI assessments. Within the CRF sample, 17 schools assessed <10% of their total student population and were therefore excluded from analyses. The PACER test was the most common CRF assessment approach, as 93% of students completed the PACER (7% completed the mile-run). Within the BMI sample, all schools collected data from >10% of students. School characteristics data were available for all schools, and academic data were available for 166 schools in the CRF sample, and 67 schools in the BMI sample, which were the final analytic samples.

### 3.1 School characteristics

The CRF sample included 166 schools from 16 different states, with an average enrollment of 505 students and relatively even distribution across cohorts 1–4 (Table 1). On average, schools served 68% of students from economically disadvantaged households, and most schools (86%) were elementary. Fewer than half (43%) of schools were in a city. Racial/ethnic composition varied, with 22% of schools serving majority White students, 16% majority Black, 33% majority Hispanic, 3% majority another race/ethnicity, and 26% having no racial/ethnic majority. The BMI sample included 67 schools across 10 states (Table 1). Characteristics of the BMI sample were like the CRF sample, with some differences: the BMI sample included a higher percentage of Cohort 3 schools, city schools (70%), schools with no racial/ethnic majority (34%), majority black (30%), and a majority another race (8%). There was also a lower percentage of schools that were majority White (12%) or majority Hispanic (16%).

**Table 1:** Descriptive statistics of U.S. school students from 2019 to 2023.

School characteristics	Cardiorespiratory fitness (*n* = 166)	Body mass index (*n* = 67)
Total Number of Students, m (SD)	505 (253)	533 (256)
Cohort, % (n)		
1	18 (30)	17 (12)
2	23 (39)	15 (10)
3	28 (46)	51 (34)
4	31 (51)	16 (11)
Economic Disadvantage, m (SD)	68 (28.7)	67 (31.2)
Title 1 Eligible, % (n)	83 (137)	79 (53)
School Level, % (n)		
Elementary	86 (143)	90 (60)
Middle/High/Mixed	14 (23)	10 (7)
Locale, % (n)		
City	43 (71)	70 (47)
Non-city	57 (95)	30 (20)
Race/Ethnicity, % (n)		
White (non-Hispanic)	22 (36)	12 (8)
Diverse***	26 (44)	34 (23)
Black	16 (26)	30 (20)
Hispanic	33 (55)	16 (11)
Another Race	3 (5)	8 (5)

Diverse schools have no Race/Ethnicity majority group; Title 1 Eligibility is based on having ≥40% of students from economically disadvantaged households.

### 3.2 School characteristics and CRF

Unadjusted mixed-effects linear regression models indicated school enrollment was positively associated with the percentage of students meeting HFZ standards for CRF, whereas economic disadvantage was inversely associated with CRF. There were differences in percentage of students meeting HFZ standards between majority Black and majority Hispanic schools, when compared to majority White schools. However, in multivariable models, only race/ethnicity was significant (Table 2). Specifically, schools serving majority Black students had significantly lower percentage of students meeting HFZ standards for CRF (b = −19.3, *p* = 0.006) compared to schools serving majority White students, after adjusting for other variables. The adjusted multilevel model had a marginal R^2^ of 0.22 and a conditional R^2^ of 0.35, indicating that the fixed effects explained 22% of the variance in the percentage of students meeting HFZ standards for CRF, while including state-level variation, explained 35%.

**Table 2:** Associations between school characteristics & FitnessGram of U.S. school students from 2019 to 2023.

School characteristics	Cardiorespiratory fitness (n = 166)		Body mass index (n = 67)	
	Unadjusted (SE)	Adjusted (SE)	Unadjusted (SE)	Adjusted (SE)
Total Number of Students	0.01* (<0.01)	0.01 (<0.01)	<0.01** (<0.01)	<0.01 (<0.01)
Economic Disadvantage	−0.23** (0.06)	−0.14 (0.10)	−0.17*** (0.03)	−0.10* (0.05)
School Level (ref: non-elementary)				
Elementary	2.5 (4.9)	5.7 (4.8)	−1.6 (3.5)	−0.3 (3.1)
Locale (ref: non-city)				
City	−2.6 (3.8)	3.6 (3.8)	−7.6 (2.1)	−2.1 (2.4)
Race/Ethnicity (ref: White)				
Diverse	−4.4 (4.9)	−3.2 (5.0)	−4.5 (2.7)	−1.9 (2.9)
Black	−23.8*** (5.9)	−19.3** (6.5)	−7.8** (2.8)	−2.2 (3.2)
Hispanic	−10.8* (4.9)	−7.3 (5.5)	−15.4*** (3.1)	−9.7** (3.7)
Other	8.1 (10.3)	10.1 (10.4)	6.6 (3.8)	0.6 (3.9)

Note: *p < 0.05, **p < 0.01, ***p < 0.001.

### 3.3 School characteristics and BMI

In bivariate linear regression models, several school characteristics (enrollment, economic disadvantage, and race/ethnicity) were associated with the percentage of students meeting HFZ standards for BMI. However, in multivariable models, only the economic disadvantage and race/ethnicity remained significant (Table 2). Specifically, serving a higher percentage of economically disadvantaged students was associated with a lower percentage of students meeting HFZ standards for BMI (b = −0.10, *p* < 0.04). Additionally, schools serving majority Hispanic students demonstrated significantly lower percentages of students meeting HFZ standards for BMI (−9.7, *p* = 0.007) compared with schools serving majority White students, after controlling for other variables. The adjusted model had an R^2^ value of 0.60, indicating 60% of the variance was explained by the model.

### 3.4 CRF, school characteristics, and academics

Bivariate mixed-effects linear regression models revealed the percentage of students meeting HFZ standards for CRF was directly associated with the percentage of students performing at or above grade-level in math and ELA. There were also indirect associations between economic disadvantage and academic outcomes, and differences in academic outcomes for several race/ethnicity categories (Table 3). In multivariable models, CRF remained significant, and directly associated with math (b = 0.10, *p* = 0.04) and ELA (b = 0.13, *p* = 0.006), when holding other variables constant. This indicates that a 10-percentage point increase in students meeting HFZ standards for CRF was associated with a 1-percentage point increase in math and 1.3-percentage point increase in ELA. Economic disadvantage remained inversely associated with math (b = −0.38, *p* < 0.001) and ELA (b = −0.42, p < 0.001) when holding other variables constant, indicating a 10-percentage point increase in economically disadvantaged students was associated with a 3.8 percentage point decrease in math and 4.2 decrease in ELA. For race/ethnicity, only majority Black and majority Hispanic schools remained significantly lower in the percentage of students at or above grade-level for math (b = −12.7, *p* = 0.007; b = −9.6, *p* = 0.03, respectively) and ELA (b = −12.3, *p* = 0.005; b = −10.8, *p* = 0.008, respectively) compared to majority White schools. Additionally, there was a non-significant trend for an inverse association between student enrollment and the percentage of students at or above grade-level for ELA (b = −0.01, *p* = 0.05), but no evidence of association with math (b < −0.01, *p* = 0.27). The marginal R^2^ for the multilevel math model was 0.52 and the conditional R^2^ was 0.70. The ELA model had a marginal R^2^ of 0.59 and a conditional R^2^ of 0.75. There was no evidence of interactions between economic disadvantage (based on Title 1 eligibility) and CRF (*p* > 0.05) for both math and ELA models.

**Table 3:** Cardiorespiratory fitness and school characteristics with academics of U.S. school students from 2019 to 2023 (n = 166).

School characteristics	Math		English language arts	
	Unadjusted (SE)	Adjusted (SE)	Unadjusted (SE)	Adjusted (SE)
Cardiorespiratory Fitness	0.30*** (0.06)	0.10* (0.05)	0.33*** (0.06)	0.13** (0.05)
Total Number of Students	<0.01 (<0.01)	<−0.01 (<0.01)	<0.01 (<0.01)	−0.01* (<0.01)
Economic Disadvantage	−0.53*** (0.04)	−0.38*** (0.06)	−0.56*** (0.04)	−0.42*** (0.06)
School Level (ref: non-elementary)				
Elementary	3.73 (4.22)	1.29 (3.07)	−0.05 (4.30)	2.73 (2.86)
Locale (ref: non-city)				
City	−3.65 (3.53)	2.83 (2.59)	−7.92* (3.53)	−0.65 (2.42)
Race/Ethnicity (ref: White)				
Diverse	−11.38** (3.54)	−2.84 (3.38)	−11.84** (3.43)	−2.02 (3.15)
Black	−29.24*** (4.44)	−12.73*** (4.68)	−32.06*** (4.31)	−12.29** (4.37)
Hispanic	−29.27*** (3.75)	−9.56*** (4.34)	−31.23*** (3.63)	−10.81*** (4.05)
Other	13.99 (7.24)	9.42 (6.77)	14.47* (7.05)	8.21 (6.30)

Note: *p < 0.05, **p < 0.01, ***p < 0.001.

### 3.5 BMI, school characteristics, and academics

In bivariate mixed-effects linear regression models, the percentage of students meeting HFZ standards for BMI was directly associated with the percentage of students performing at or above grade-level in math and ELA (Table 4). There was also evidence of associations with economic disadvantage, locale, and race/ethnicity. However, in multivariable models, the relations between BMI, and math (b = 0.26, *p* = 0.25) and ELA (b = 0.18, *p* = 0.47) were no longer statistically significant, while associations with economic disadvantage, school-level, and race/ethnicity remained significant. Economic disadvantage was inversely associated with ELA performance (b = −0.34, *p* < 0.001) when holding other variables constant, indicating a 10-percentage point increase in economically disadvantaged students served was associated with a 3.4 percentage point decrease in ELA achievement. The association between economic disadvantage with math did not reach statistical significance (b = −0.18, *p* = 0.05) in the BMI sample. Elementary schools demonstrated a significantly higher percentage of students at or above grade-level for math (b = 17.31, *p* = 0.001), but the association with ELA was not statistically significant (b = 9.9, *p* = 0.08). For race/ethnicity, schools serving majority Black students had a significantly lower percentages of students at or above grade-level for math (b = −16.9, *p* = 0.003) and ELA (b = −15.1, *p* = 0.012) compared with majority White schools. Schools serving majority students of another race had significantly higher percentage of students at or above grade-level for math (b = 16.1, *p* = 0.02) and ELA (b = 16.7, *p* = 0.03) compared to majority White schools. Majority Hispanic schools had lower percentages of students at or above grade-level for math (b = −14.5, *p* = 0.06) and ELA (b = −10.1, *p* = 0.17), although the associations were not statistically significant. The adjusted multilevel math model had a marginal R^2^ value of 0.54 and a conditional R^2^ of 0.80, whereas the adjusted multilevel ELA model had a marginal R^2^ value of 0.71 and a conditional R^2^ of 0.73. There was no evidence of interactions between economic disadvantage (based on Title 1 eligibility) and BMI (*p* > 0.05) for both math and ELA models.

**Table 4:** Body mass index and school characteristics with academics of U.S. school students from 2019 to 2023 (n = 67).

School characteristics	Math		English language arts	
	Unadjusted (SE)	Adjusted (SE)	Unadjusted (SE)	Adjusted (SE)
BMI	0.98*** (0.21)	0.26 (0.23)	1.07*** (0.23)	0.18 (0.25)
Total Number of Students	<0.01 (<0.01)	<−0.01 (<0.01)	0.01 (0.01)	<−0.01 (<0.01)
Economic Disadvantage	−0.44*** (0.06)	−0.18 (0.09)	−0.51*** (0.06)	−0.34*** (0.09)
School Level (Referent: non-elementary)				
Elementary	13.26 (6.78)	17.31** (4.99)	4.95 (7.66)	9.93 (5.62)
Locale (Referent: non-city)				
City	−16.55*** (4.62)	−0.41 (4.25)	−19.78*** (4.93)	0.06 (4.40)
Race/Ethnicity (Referent: White)				
Diverse	0.04 (5.59)	−2.16 (5.17)	0.36 (5.73)	0.37 (5.87)
Black	−19.30** (5.95)	−16.85* (5.62)	−23.00*** (6.08)	−15.06* (5.97)
Hispanic	−16.15* (6.83)	−14.52 (7.58)	−17.20* (6.79)	−10.13 (7.40)
Other	24.03** (7.69)	16.11* (7.03)	27.88** (8.11)	16.72* (7.48)

Note: *p < 0.05, **p < 0.01, ***p < 0.001.

## 4 Discussion

This study examined school-level associations between school characteristics, health-related fitness, and academic performance. Our findings revealed economic disadvantage was inversely associated with percentage of students meeting HFZ BMI standards, and majority Black and majority Hispanic schools had lower percentages of students meeting HFZ standards for CRF and BMI, respectively, compared to majority White schools. We also observed inverse associations between economic disadvantage and academic performance, along with differences in academic performance across schools with different racial/ethnic compositions. Importantly, school-level CRF was directly associated with academic performance. There were no significant associations between healthy BMI and academic performance. Collectively, these findings suggest there are key differences in both fitness and academic achievement across school communities, and a link between school-level CRF and academic performance.

Existing research highlights disparities in health-related fitness and academic outcomes across socioeconomic status (SES) and racial/ethnic groups. Children from higher SES families demonstrate better fitness compared to peers from lower SES backgrounds (Bai et al., 2016; Wolfe et al., 2020), and students attending schools with a greater concentrations of economic disadvantage have lower odds of meeting HFZ standards than students in schools serving more affluent communities (Walker et al., 2020). Similar disparities are documented for race and ethnicity, with Black and Hispanic children showing lower fitness levels compared to White children (Bowser et al., 2016; Konty et al., 2020). These patterns also exist in academic performance, as schools serving predominantly low-SES, Black, or Hispanic students consistently report lower academic outcomes than high-SES or majority White schools (White et al., 2016; Sirin, 2005). Our study adds to this evidence, highlighting the health and educational inequities facing children in economically disadvantaged, minoritized communities.

Low fitness levels in under-resourced and minoritized communities are likely due to multiple factors. First, research has demonstrated that school-based physical activity opportunities (e.g., PE, recess, classroom-based PA) are inadequately implemented in schools serving low-income or minoritized student populations, likely contributing to lower fitness levels (Craig et al., 2024; Turner and Chaloupka, 2017; Carlson et al., 2014). Second, children in these communities often have financial barriers and limited access to safe recreational spaces, which contribute to fewer physical activity opportunities outside of school (McKenzie et al., 2013; Chang and Kim, 2017). The lack of physical activity and lower fitness levels are especially concerning given the evidence linking fitness to academic achievement (Basch, 2011).

Growing evidence supports the connection between fitness and academic outcomes, both at the individual- and school-levels (Santana et al., 2017; Álvarez-Bueno et al., 2020; Zhu et al., 2022; Castelli et al., 2007; Welk et al., 2010). Our study further highlights disparities in fitness and academic performance across a sample of schools from 16 different states, and suggests a school-level link between fitness and academics. These findings are valuable for district leaders and policymakers, as they suggest equitable access to school-based physical activity opportunities that improve fitness may help support academic achievement. Such efforts are important given current trends prioritizing additional instructional time at the expense of recess, physical education, and other physical activity opportunities (Committee on Physical Activity and Physical Education in the School Environment, Food and Nutrition Board, Institute of Medicine, 2013). Additional instruction time may have diminishing returns given students movement needs and the evidence linking physical activity and fitness to academic achievement.

This are study limitations to consider. First, this was an observational study using secondary data, which restricts the ability to draw causal inferences between fitness and academic outcomes. Second, the sample included schools in the NFL PLAY60FG program, which was mostly elementary schools in the NFL markets. Also, schools received resources for fitness promotion, potentially limiting generalizability. Third, there was variation in the proportion of students completing FitnessGram assessments within schools, which may have resulted in some schools' data not fully representing their student populations. Additionally, differences in academic assessment systems across states could have introduced variability affecting findings. Lastly, this study did not control for potential confounding variables such as physical activity opportunities (both in and out of school) and other factors impacting support for physical activity in schools and communities (e.g., school culture, family support). Future work should further examine school-level associations between fitness and academics while accounting for these factors and explore the mechanisms driving relations.

Despite limitations, this study had strengths. The study used a sample of schools across 16 states, enhancing the generalizability of findings. We also merged data from multiple sources, including the National Center for Education Statistics for school characteristics, state departments of education for academic data, and NFL PLAY 60 FG for fitness data. This led to a unique and comprehensive dataset for analyses and allowed for simultaneously examining associations between key school-level characteristics, fitness, and academic outcomes. Further, the school level analysis has practical relevance for district and school decision-makers.

## 5 Conclusions

Findings revealed disparities in school-level fitness associated with economic disadvantage and race/ethnicity, highlighting a need for physical activity opportunities in schools serving these communities. There were also differences in academic performance based on economic disadvantage and race/ethnicity. After controlling for economic disadvantage and race/ethnicity, there was a significant direct association between CRF and academic achievement, emphasizing the independent contribution of CRF on students' academics. These findings reinforce the importance of prioritizing fitness promotion in schools serving economically disadvantaged and racially/ethnically diverse communities, and to recognize physical activity and fitness as integral strategies for supporting health and academics.

## CRediT authorship contribution statement

**Timothy J. Walker:** Writing – original draft, Methodology, Formal analysis, Conceptualization. **Andjelka Pavlovic:** Writing – review & editing, Methodology, Conceptualization. **Christopher D. Pfledderer:** Writing – review & editing, Methodology, Formal analysis, Data curation. **Derek W. Craig:** Writing – review & editing, Methodology. **Kevin Lanza:** Writing – review & editing, Methodology. **Kempson Onadeko:** Writing – review & editing, Formal analysis, Data curation. **Matthew Lee:** Writing – review & editing. **Laura F. DeFina:** Supervision.

## Funding

We would like to acknowledge the support provided to the Kenneth H. Cooper Institute at the Texas Tech University Health Sciences Center funded by the 10.13039/100006425National Football League Foundation. This work was partially supported by the Center of Health Promotion and Prevention Research.

## Declaration of competing interest

The authors declare that they have no known competing financial interests or personal relationships that could have appeared to influence the work reported in this paper.

## Data Availability

Data will be made available on request.
